# What Are the Determinants of a Workplace Health Promotion? Application of a Social Marketing Model in Identifying Determinants of Physical Activity in the Workplace (a Qualitative Study)

**DOI:** 10.3389/fpubh.2020.614631

**Published:** 2021-01-14

**Authors:** Mohammad Hossein Kaveh, Mehdi Layeghiasl, Mahin Nazari, Leila Ghahremani, Masoud Karimi

**Affiliations:** ^1^Research Center for Health Sciences, Institute of Health, School of Health, Shiraz University of Medical Sciences, Shiraz, Iran; ^2^School of Health, Shiraz University of Medical Sciences, Shiraz, Iran

**Keywords:** physical activity, workplace health promotion program, social marketing, qualitative study, directed content analysis

## Abstract

**Background and objective:** Physical activity is an important behavior to reduce the risk of non-communicable diseases. Providing the context for physical activity in the workplace in addition to promoting the employees' physical and mental health has significant economic benefits for organizations. We conducted the present study using a social marketing model to explain the determinants of a Workplace Health Promotion in promoting physical activity among employees of government organizations in Yasuj, Iran.

**Materials and methods:** The present study was qualitative research with directed content analysis based on the social marketing model. Thirty-three employees of government organizations in Yasuj were included in the study using the purposive sampling method. The data collection method included semi-structured interviews and observation. Data analysis was performed manually and by the qualitative content analysis method. The implementation data were systematically sorted and analyzed and classified into five steps.

**Results:** Organizational structure, organizational policies, and a supportive interpersonal climate were extracted and categorized as characteristics of workplace health promotion programs from participant interviews. In addition to participants' emphasis on receiving information from reputable sources, virtual communication networks such as WhatsApp and real communication networks such as physicians and specialists were their preferred media for education and information.

**Conclusion:** Due to the complexity of workplace, diversity, and multiplicity of factors and determinants of physical activity, the findings of the present study will be a basis for designing an appropriate and effective intervention and organizational changes to promote physical activities among employees in the future.

## Introduction

Non-communicable diseases account for 72.3% of all deaths and are the leading causes of death worldwide (1). Physical activity is an important behavior to reduce risk of these diseases (2). The fourth leading risk factor for death worldwide is inadequate physical activity (3). However, paying attention to “health” in everyday environments can be a way to improvement the individual health. “The individuals' health is formed in their daily living environment where they learn, work, and play” (4). In 1986, the creation of health supportive environments (environment-based approaches) was introduced as a strategy of the Ottawa Charter (5). In recent decades, this strategy has shifted to health promotion environments with an aim to change from personal risk factors and control physical harmful factors in the workplace (6). Workplace health promotion programs are comprehensive programs that can be implemented with an aim to adopt healthy behaviors, improve the individual health, and reduce the prevalence of chronic diseases (7).

A high percentage of the active population of society is present in the workplace and they are easily accessible (6). These people also spend half of their time in the workplace (8). On the other hand, a healthy and productive workforce is essential to improve the population health and economic growth (9, 10). Therefore, changing the workplace to a suitable supportive environment is a proposed solution to health promotion. However, many studies have reported a low participation in this field (2). Various variables and determinants affect participation in physical activity programs and its continuity and cannot be explained and predicted based on a variable. Understanding the audience's views and opinions about barriers and facilitators affecting the acceptance and continuity of behavior are important determinants of physical activity behavior (11).

The existing theoretical frameworks for behavior analysis and change are good guides for effective planning and intervention in the health education and promotion. Health promotion programs, which are based on theories and models, lead to useful and effective results (12). Social marketing is used as a model for increasing the benefits, reducing the barriers, providing the motivation or change the opportunities for a particular behavior (13). Social marketing seeks to provide the effectiveness and improve the likelihood of choosing behavior by people in society and its continuity. Communication needs, media habits, different tendencies, social, cultural and structural, or environmental factors, which have a positive or negative impact on audience behavior, are identified in social marketing by the help of participatory research. The analysis of audience before designing effective intervention strategies with the aim of better understanding of needs, health problems and their determinants is a key feature of the social marketing model (14). The present qualitative research aimed to examine the government employees' needs, interests and views about physical activities based on the marketing mix (4p), including four main structures (product, price, place, and promotion), and achieve a physical activity program with high acceptability in the workplace.

Numerous qualitative studies have been conducted using the principles of social marketing (15, 16). Studies have also used social marketing models to promote physical activity (3, 17, 18), but our studies indicated no qualitative study on the directed content analysis based on the social marketing model in creating a Workplace Health Promotion with an aim to promote physical activity among government employees in the world.

## Materials and Methods

The present study was a qualitative research with a directed content analysis type based on the social marketing model. In the directed content analysis, the existing theories or results of previous studies form the basis of analysis. We used the present method since providing more descriptions of existing theories or conceptual expansion of a theoretical framework is an application of a directed content analysis (19). The reason for applying a qualitative method was its capabilities in creating a deep insight into the individuals' perceptions and experiences (20), helping to gain a new perspective on issues that even have some knowledge about them, and also achieving subtle details of phenomena that are difficult to express in a quantitative way (21).

### Participants and Sampling Method

The participants were 33 employees of government organizations in Yasuj. We used a purposive sampling method in the present study (22). Data collection process was continued until saturation of each concept was reached, and further data collection failed to contribute new information. The sampling was then completed with data saturation (23). The purpose of selecting samples was their inclusion according to a specific status and inclusion criteria, including: (1) employees of organizations with at least 50 employees, (2) employees of specific, contractual and formal contracts of organizations, and (3) having more than a year of work experience in public offices and organizations of Yasuj. Also, all participants were physically healthy. Participants in this study were selected based on two criteria of experimental fit and good informant, which is called the quality of the participant. Also, the willingness of the participants was considered as a principle (24). The selection of samples was done with an active presence of the researcher in organizations (25). We listed government offices and selected Electric Power Distribution Company, Department of Education, the University of Medical Sciences and Economic and Financial Affairs Directorate General. In this regard, the researcher attended government offices in Yasuj and invited employees to participate in the interview. The interview was conducted if desired and after completing the informed consent form.

### Instrument and Data Collection Method

The data collection method of research consisted of the semi-structured interviews and observation. For the semi-structured interviews, a series of general questions were pre-designed based on the social marketing mix using a panel of experts and previous studies. Some in-depth questions were asked during the interview to find out the depth of participants' perspectives. Other questions came to the researchers' minds during the analysis of the interviews and they were used in subsequent interviews (26). Accordingly, the interview guide was designed based on 4p marketing mix. For example for the product: What features and characteristics would you like physical activity programs to have in your workplace? Price: In your opinion, if you want to have daily and appropriate physical activity at work, what barriers and problems do you face? Place: do you think are What places more suitable for Run physical activity programs? Promotion: How do they communicate with you (what channel, what method, who) to talk about physical activity? Interviews were recorded with the informed consent of the participants. During the observation, the participants' feelings, mood, and behavior were taken into consideration and noted.

### Data Analysis

We manually analyzed data using the qualitative content analysis method. Immediately after the interviews and observations, the recorded text and notes were typed word by word. The files, along with the notes during the interviews were read for several times carefully with an aim to gain a general understanding of the text (27). Data collection continued until the end of the study. Researcher simultaneously summarized and analyzed data. These processes continued regularly until the data saturation (23).

We systematically classified, sorted and analyzed data in several steps. In the first step, the same original text was a meaning unit without manipulation. In the second step, we summarized the main text and put in Condensed meaning units while preserving the meaning. The third step usually led to encoding one or two codes with one or two long words. In fact, it was a label code that explained exactly what the particular condensed unit meant. Step four included the category and consisted of grouping codes that were relevant in terms of content or text. In other words, the codes were classified into various aspects, similarities or differences between the text content. The final step covered the theme indicating the original meaning and hidden content in two or more categories (27). In this way, latent themes and patterns revealed within the data content of the participants (28). The classification was done based on similarities, and we then placed the classes as 4p subcategories social marketing mix (product, price, place, and promotion) as fundamental themes (3). The research team sought to create the intra-category homogeneity and more inter-category heterogeneity.

### Trustworthiness of Data

We utilized the triangulation and member check to achieve the credibility, the External Check to achieve the Dependability, the peer check to achieve the Confirmability, and expression of research limitations and comparison of findings from a participant to another during the research to achieve the Transferability (29, 30).

## Results

The results of this study show the needs, interests and views of government employees about physical activity based on the marketing mix. The main findings of the study and a summary of these findings are reported below.

Thirty-three employees of government organizations participated in the present study. Table 1 presents the participants' demographic characteristics.

**Table 1:** Participants' demographic characteristics.

**Age**	**Education level**	**Sex**	**Marital status**
25–34 (*n* = 6) 35–44 (*n* = 14) 45–54 (*n* = 9) 55 and over (*n* = 4)	Under high school diploma (*n* = 8) High school diploma (*n* = 2) Academic (*n* = 23)	Male (*n* = 20) Female (*n* = 13)	Married (*n* = 30) Single (*n* = 3)

Findings of the data analysis were categorized based on the 4p marketing mix (product, price, place, and promotion).

### Product

Since our product was “physical activity” behavior among employees of governmental organizations we investigated the benefits and advantages of physical activity based on the audience's interest to make the behavior more attractive and valuable so that it would lead to the acceptance of the proposed product by a target audience. To achieve this goal, we identified and categorized the most important benefits of the product (physical activity behavior) along with the audience's needs.

Therefore, the physical activity characteristic as a desired product is an important determinant of the acceptance and continuity of behavior. Three groups, namely the supporting organizational structure, supporting organizational policies, and supporting interpersonal atmosphere were Main findings extracted and categorized from the participants' interviews. Table 2 summarizes the main findings.

**Table 2:** Product (Product characteristics from participants' perspective).

**Participant**	**Meaning unit**	**Condensed meaning units**	**Code**	**Category**	**Theme**
38 years old Female	The coordinator is very important. There must be someone in the organization for constant follow of the presence of employees and the assistance of managers.	Someone who follows the presence of employees and helps the managers	Determining a person responsible for promoting the employees' health	Supportive organizational structure	Product
29 years old male	I think considering a single and independent organization responsible for employees' physical activity and health is very helpful.	Considering an independent unit responsible for employees' physical activity and health	The staff health promotion unit		
32 years old male	In my opinion, cheerfulness is the most important issue that is never taken into consideration. When the atmosphere is lively, colleagues go to exercise more together. For example, holding various competitions and creating entertainment such as competitions like darts, ping pong and chess cause a joy among employees and can greatly increase physical activity.	In my opinion, cheerfulness is the most important issue. It can greatly increase physical activity.	Creating a happy environment		
38 years old male	Physical activity should be considered in office time and in the office because more people participate.	Physical activity should be considered in office time and in the office.	Proper time	Supportive organizational policies	
30 years old male	A favorite activity should be considered. Our colleagues may be interested in other sports that we do not. Some are older or even overweight and need to do some proper exercise. Many friends may not be interested in swimming. In my opinion, we should see the employees are interested in what sport and where they like to go for exercise, and consider the same place for them.	Favorite activities should considered for a person.	Organizational motivation		
49 years old male	Employees' health should be important for the manager and have a plan for it. For example, in a manager selection interview, I should check to see if this person who wants to be a manager has a plan for the employees' health, or the manager does not pay attention to their health.	Employees' health should be important for the manager and that program.	Organizational priorities		
29 years old male	Creating a friendly environment is also effective. If it is suggested by people who are closer to me, I will participate, even when I did not want to, but at the invitation of my close friends, I canceled the rest of work and went to sports.	Creating a friendly environment is effective. I went to sport after invitation by my close friends.	Friendly atmosphere	Interpersonal supportive atmosphere	
28 years old male	Happy and cheerful individuals should be employed in organizations so that others can participate in the programs.	Happy and cheerful individuals should be employed in organizations.	Encouraging peer		

### Price

To achieve this aim, the perceived costs or barriers to behavior should be identified from perspective of the audience. Before deciding to adopt a behavior, the audience contrasts perceived costs with perceived benefits of the behavior and adopts behaviors with prices and costs less than the benefits. The Main findings of this section were divided into two general categories: benefits and barriers. Then, the benefits were classified into personal and organizational benefits; and barriers into three groups: personal, inter-personal, and organizational barriers. Table 3 summarizes the main findings.

**Table 3:** Price (barriers and costs of conducting behavior from participants' perspective).

**Participant**	**Meaning unit**	**Condensed meaning units**	**Code**	**Category**	**Theme**
32 years old male	Mental barrier is a kind of barriers. The person is bored to do sports and does not want to go. This makes the person not interested in doing physical activity.	Person is bored to do physical activity.	Lack of motivation	Personal barriers	Price
29 years old male	I think spouses are main causes of physical inactivity. They do not cooperate in any way, for example, they make programs that their spouses can no longer go for a walk.	Spouses are main causes of physical inactivity	Lack of cooperation of spouses	Interpersonal barriers	
42 years old male	In my opinion, The 45-min law of morning exercise is the worst plan because first you have to come early in the morning to point fingers and then go to exercise, and it is worse that you have to come in formal clothes and then no one exercises in formal clothing, and no one goes to office in sportswear in the morning.	The 45-min law of morning exercise is the worst plan because you have to go to office wearing tuxedos, and nobody exercises in formal clothing.	inappropriate organizational planning	Organizational barriers	
38 years old male	Considering points for employment of those who have adequate physical activity. In fact, if we want employees to be physically active, they should be taken into consideration in the organization.	Considering points for employment of those who have adequate physical activity	Supportive evaluation system of the organization	Organizational benefits	
33 years old male	The office should provide necessary facilities for the person. If there are bathroom and changing room in the office, the employees go to physical activity, and then take a shower and change their clothes.	The office should provide necessary facilities for the person.	Organizational support		
39 years old female	In fact, we are at work until two and a half o'clock, and then it is evening and usually everyone is married and they do not have enough time to participate. If it is in office time like the morning for a little walking at a closer place to the office, more employees participate.	In my opinion, if it is in office time is better, and its place should be close to the office, so more employees attend	The right time and place		
31 years old female	The individuals play main roles for their health, If they value their own health, they do exercise even if they take a leave.	If individuals value their health, they go to sports.	Paying attention to health	Personal benefits	

#### Place

To achieve this goal, we identified and categorized the audience's desired and interesting places for physical activity, and receiving information and messages. Table 4 summarizes the main findings. Participants suggested the place of training classes and physical activity near the workplace. Based on Main findings, the most important issue was the audience's access to a suitable place (proper space).

**Table 4:** Place (place of behavior and receiving educational interventions from the participants' perspective).

**Participant**	**Meaning unit**	**Condensed meaning units**	**Code**	**Category**	**Theme**
39 years old Female	The same office environment and halls are suitable for classes and programs.	The office environment and halls are suitable for classes and programs	Workplace	Access (proper space)	Place
32 years old male	I have not attended any classes, but I had studies, and I think it is better and we are more comfortable in our office.	I think we are more comfortable in our office.			
39 years old male	The closest places to the workplace are the most suitable places for physical activity.	The nearest places are suitable for physical activity.	Close to workplace		
42 years old male	I think a suitable place near our workplace is much more suitable for our exercise in terms of saving time.	Close space is more suitable for sports.			

#### Promotion

The method of establishing a relationship with the audience was identified to achieve this goal. The media, advertising and other methods to reach consumers were examined to inform and encourage them. Three groups of message boosters, real communication networks, and virtual communication channels were extracted and categorized as the main findings of the participants' interviews. Table 5 summarizes the main findings.

**Table 5:** Promotion (the way of promoting behavior from participants' perspective).

**Participant**	**Meaning unit**	**Condensed meaning units**	**Code**	**Category**	**Theme**
50 years old male	The presence of sports managers and enthusiasts is very good. An athletic and sports-minded manager can institutionalize sports in the organization because they have an influence on employees and their presence causes more employees to participate in the physical activity program; hence, managers should be justified and help.	The presence of sports managers and enthusiasts causes more employees to attend the physical activity program because they have an influence on employees.	Managers' participation	Boosting messages	Promotion
26 years old female	The manager and public relations of the organization are very important for trainings and their words are given more attention.	The manager and public relations of the organization are very important for training.	Influential educational media		
38 years old male	Using the capacity of the media like video media, and using the language of art like a one-minute clip have more impact than thousands of media.	Video media. A one-minute clip is effective.	Video educational media	Virtual communication networks	
34 years old Female	Cyberspace is very important, especially if they have a WhatsApp group and receive messages from a source that has scientific; and it is very effective to put the accurate information inside the group.	WhatsApp group and messages should be derived from a scientific and precise source, and should be put them inside the group.	Reliable educational media		
30 years old male	In my personal opinion, cyberspace is suitable, but provided that, for example, close and friendly people are form separate groups and work, that is, all individuals in the organization are divided into groups who are more intimate and friendly with each other and can do more exercise together.	Cyberspace is suitable. The individuals can be divided into groups that are friends and are more likely to exercise together.	Interactive educational media		
31 years old male	In my opinion, the specialists and professors in this subject are very good, I mean the doctors.	In my opinion doctors are good.	Reliable educational media	Real communication networks	
30 years old Female	Effective and dominant people or experts have experience in sports.	Experts with experience in sports	Reliable educational media		
28 years old male	Small booklets should not be bulky or overly specialized.	Small booklets for messages	Written educational media		
36 years old male	Sometimes it is very effective if there is someone who speaks interestingly and is in my own gender and tells me about self-experiences.	If someone is in my own gender, it is very effective.	peer educational media		

## Discussion

The workplace is complex and satisfying the needs of all employees will be a challenge. Individuals within the workplace have different values and barriers that keep them from being physically active. Social marketing is an approach to alleviate the barriers and increasing the benefits of behavior to encourage physical activity engagement in the workplace. In the present study, we sought to explain the components affecting the promotion of physical activity in the workplace among the Iranian government employees based on the social marketing model. A supportive organizational structure and supporting organizational policies were the most important findings of the present study and were introduced as features of physical activity program in the workplace for its acceptance, adoption and continuation by the participants. Results obtained in a study by Jørgensen et al. also indicated that workplace characteristics were important determinants of employee participation in workplace health promotion programs (2). The creation of a happy and lively environment was another effective feature in terms of the participants' views in the workplace physical activity program in the present study. Davey et al. also confirmed that it would be better to have a pleasurable place for more participation (31). According to another finding of the present study, a more attractive product (physical activity program) can be predicted for more employee participation by considering the features suggested by the participants. Studies have also found that the attractiveness of programs is effective in increasing the employee participation (32).

Findings of the present study indicated that paying attention to the prediction of diverse programs tailored to employees' conditions was a way of success in implementing the health promotion programs in the workplace. This issue has been considered in another study (31). For example, participants of the present study suggested the type of physical activity appropriate to their age for greater participation. Similar studies have found that age is an important predictor of the individual participation in physical activity programs. For instance, findings of some studies indicated that older employees were more involved (33, 34) and another study indicated that younger people were more involved in physical activity programs (33). Thus, Contrary to the reports of some studies that age was not significantly related to the level of participation in physical activity (35, 36), according to the results of the present study, many participants reported that considering the type of physical activity appropriate to the age conditions of individuals can increase participation in physical activity programs. This discrepancy may be related to differences in the type of studies, the setting and the research sample. According to findings of our study, differences in results of these studies may be due to the mismatch of the proposed activities and programs with the individuals' age. Participants believed that the program time should be in the office time and by taking into account the appropriate time. Findings from other studies also confirmed the appropriate time for implementation of the health promotion programs (2).

The results of the present study indicated that most of participants considered the personal, interpersonal and organizational barriers as the main barriers to participation in physical activity. Results of other studies indicated that those, who perceived fewer barriers to physical activity programs, were more involved (37). Consistent with the present study, another study considered the low level of personal motivation as a barriers to participation (38). Low commitment and managerial participation were among the barriers to the success of physical activity programs in the workplace in the present study; and most participants considered them as important barriers. A study also indicated that strong managerial commitments played important roles in increasing participation (39).

Other studies have introduced social support as an effective component in high employee participation in programs (40, 41). The present study also found that a supportive interpersonal atmosphere was an effective component in continuing to participate in programs. Proper access was another determinant of the employee participation in health promotion programs of the workplace. The finding was also presented in a study aimed at investigating the reasons for individuals' refusal to participate in health promotion programs in the workplace (39).

## Conclusion

Given the complexity of the workplace conditions, the diversity and multiplicity of factors and determinants of physical activity, the importance of effective interventions, and the participation of employees and managers require the use of an audience-oriented model such as social marketing. Therefore, findings of the present study will be a basis for designing an appropriate and effective intervention with an aim to promote physical activity among employees of organizations in the future.

### Research Limitations

The findings of this study, similar to other qualitative studies, have little generalizability, especially since this study was conducted only on a limited number of employees. Therefore, generalization of the results of this study to other areas should be done with caution. Given that the work environment is extremely complex, there may be many other barrier that we have not addressed. for example Low self-efficacy as an individual barrier was an important issue that was not addressed in this study. Not considering the participation of organizations managers in interviews was another limitation. As is the case in qualitative studies, the number of participants was relatively small, but all participants were experienced and knowledgeable and saturation was reached for all concepts. Also, since this study was conducted regardless of the previous physical activity status of individuals, it is suggested that in future studies, physically active and sedentary individuals be separated.

## Data Availability Statement

The original contributions presented in the study are included in the article/supplementary materials, further inquiries can be directed to the corresponding author/s.

## Ethics Statement

The studies involving human participants were reviewed and approved by the executive phase of the research was conducted after approving the proposal and receiving a code of ethics (IR.SUMS.REC.1398.1208) from the ethics committee of Shiraz University of Medical Sciences. The patients/participants provided their written informed consent to participate in this study.

## Author Contributions

MHK and ML: research concept, research methodology, collecting material, statistical analysis, interpretation of results, and references. MN: research methodology, collecting material, statistical analysis, and interpretation of results. LG: research methodology, collecting material, and statistical analysis. MK: research concept, research methodology, interpretation of results, and references. All authors contributed to the article and approved the submitted version.

## Conflict of Interest

The authors declare that the research was conducted in the absence of any commercial or financial relationships that could be construed as a potential conflict of interest.
